# Correlation of thyroid hormone measurements with thyroid stimulating hormone stimulation test results in radioiodine‐treated cats

**DOI:** 10.1111/jvim.15909

**Published:** 2020-10-12

**Authors:** Jennifer Wakeling, Teresa Hall, Timothy L. Williams

**Affiliations:** ^1^ Douglas College Coquitlam British Columbia Canada; ^2^ North West Nuclear Medicine Vancouver British Columbia Canada; ^3^ Department of Veterinary Medicine University of Cambridge Cambridge United Kingdom

**Keywords:** blood pressure, chronic kidney disease, feline, free thyroxine, hypertension, triiodothyronine., TSH

## Abstract

**Background:**

Iatrogenic hypothyroidism can develop after radioiodine‐I^131^ (RAI) treatment of hyperthyroid cats and can be diagnosed using the thyroid stimulating hormone (TSH) stimulation test.

**Objectives:**

To assess the effect of noncritical illness on TSH stimulation test results in euthyroid and RAI‐treated cats. To assess the correlation of low total‐thyroxine (tT4), low free‐thyroxine (fT4), and high TSH concentrations with TSH stimulation test results.

**Animals:**

Thirty‐three euthyroid adult cats and 118 client‐owned cats previously treated with RAI.

**Methods:**

Total‐thyroxine, fT4, and TSH were measured, and a TSH stimulation test was performed in all cats. Euthyroid control cats were divided into apparently healthy and noncritical illness groups. RAI‐treated cats were divided into RAI‐hypothyroid (after‐stimulation tT4 ≤ 1.5 μg/dL), RAI‐euthyroid (after‐stimulation tT4 ≥ 2.3 μg/dL *OR* after‐stimulation tT4 1.5‐2.3 μg/dL and before : after tT4 ratio > 1.5), and RAI‐equivocal (after stimulation tT4 1.5‐2.3 μg/dL and tT4 ratio < 1.5) groups.

**Results:**

Noncritical illness did not significantly affect the tT4 following TSH stimulation in euthyroid (*P* = .38) or RAI‐treated cats (*P* = .54). There were 21 cats in the RAI‐equivocal group. Twenty‐two (85%) RAI‐hypothyroid cats (n = 26) and 10/71 (14%) of RAI‐euthyroid cats had high TSH (≥0.3 ng/mL). Twenty‐three (88%) RAI‐hypothyroid cats had low fT4 (<0.70 ng/dL). Of the 5 (7%) RAI‐euthyroid cats with low fT4, only one also had high TSH. Only 5/26 (19%) RAI‐hypothyroid cats had tT4 below the laboratory reference interval (<0.78 μg/dL).

**Conclusions and Clinical Relevance:**

The veterinary‐specific chemiluminescent fT4 immunoassay and canine‐specific TSH immunoassay can be used to aid in the diagnosis of iatrogenic hypothyroidism in cats.

**Abbreviations**
BUN: blood urea nitrogen
CKD: chronic kidney disease
DVM: Doctor of Veterinary Medicine
ED: equilibrium dialysis
FIV: feline immunodeficiency virus
fT4: free‐thyroxine
RAI: radioiodine I^131^
rhTSH: recombinant human TSH
RI: reference interval
SBP: systolic blood pressure
T3: total tri‐iodothyronine
TSH: thyroid stimulating hormone
tT4: total‐thyroxine
UPCR: urine protein‐creatinine ratio
USG: urine‐specific gravity

## 1 INTRODUCTION

Biochemical evidence of iatrogenic hypothyroidism in radioiodine I^131^ (RAI) treated cats has been documented for decades. Historically, iatrogenic hypothyroidism was not thought to be a clinically important problem in most cats, and treatment was not recommended unless clinical signs consistent with hypothyroidism were present.^1^ Chronic kidney disease (CKD) is a common problem in the senior cat population,^2^, ^3^ and azotemia can be worsened by treatment of hyperthyroidism, because of the decline in glomerular filtration rate that occurs after normalization of thyroid hormone concentrations.^4^ Iatrogenic hypothyroidism in cats treated with antithyroid medication and RAI increases the risk of azotemia and decreases survival time in azotemic‐treated cats^5^, ^6^ and therefore the importance of diagnosis and treatment of iatrogenic hypothyroidism is increasingly recognized.^7^

The diagnosis of iatrogenic hypothyroidism can be challenging due to the effect of concurrent illness on serum free thyroxine (fT4) and serum total thyroxine (tT4) concentrations,^8^, ^9^ the poor sensitivity and specificity of commercially available thyroid stimulating hormone (TSH) assays, none of which are specific to cats,^10^ and the variability in performance of various fT4 assay methodologies.^11^, ^12^, ^13^ Few studies have looked at the sensitivity and specificity of the available thyroid hormone analyses for the diagnosis of iatrogenic hypothyroidism in cats.^6^, ^13^

Traditionally tT4 has been used alone in cats to diagnose thyroid disease; however, tT4 has poor sensitivity and specificity for the diagnosis of iatrogenic hypothyroidism in this species,^6^ especially when laboratory reference intervals (RIs) are used. In humans and dogs, combinations of fT4, tT4, and TSH are used to confirm a diagnosis of hypothyroidism, or, if the diagnosis cannot be confirmed by measurement of single thyroid hormones, thyroid scintigraphy, TSH stimulation test or thyroid biopsy are performed.^14^

Thyroid scintigraphy is currently used as the reference test for the diagnosis of hypothyroidism in RAI‐treated cats.^6^, ^13^ Increased serum TSH concentrations (using canine‐specific chemiluminescent TSH immunoassay) are common in hypothyroid cats, whereas fT4 (using an equilibrium dialysis [ED] method) and tT4 concentrations are within laboratory RI in 75% and 46% of hypothyroid cats, respectively.^6^ Subclinical hypothyroidism, defined as cats with elevated serum TSH concentration and fT4 or tT4 within the RI, is therefore common in RAI‐treated cats. This condition is clinically important due to the potential harmful effects on renal function and consequent decrease in survival time.^6^

The TSH stimulation test is considered a reliable reference test for hypothyroidism in healthy dogs that are not on thyroid suppressive medications (eg, glucocorticoids).^15^, ^16^ Test criteria for the TSH stimulation test in cats using recombinant human TSH (rhTSH) were derived using 7 to 8 month old, specific pathogen free, research cats which are not representative of the typically senior population of client‐owned cats with iatrogenic hypothyroidism.^17^ There are limited data published looking at the response of mature adult, or senior cats to the exogenous administration of rhTSH.^18^, ^19^

In this prospective study, mature adult cats were recruited as a control group and to assess the effect of noncritical illness on the response of mature adult euthyroid cats to TSH administration. Radioiodine I^131^‐treated cats presenting for wellness examinations were recruited to receive a TSH stimulation test as a reference test of thyroid function, to test the correlation of low tT4, low fT4, and high TSH with the results of the TSH stimulation test.

## 2 METHODS

### 2.1 Control cats

Mature adult cats were recruited from a local shelter and from study staff, as control cats for the study. Cats on thyroid‐suppressive medications (eg, prednisolone, budesonide, chlorambucil, tricyclic antidepressants, or fluoxetine) and cats diagnosed with hyperthyroidism were excluded. Each control cat received a physical examination, systolic blood pressure (SBP) measurement, routine biochemistry and hematology, urinalysis and urine protein : creatinine ratio (UPCR), measurement of tT4, fT4 and TSH concentrations, and a TSH stimulation test (see below). Weight at the time of the visit was compared to historical shelter records or the patient's previous medical record. Additional tests were performed as needed and available on the day, such as tonometry, ECG, radiography, and cytology. Follow‐up diagnostic testing (such as biopsy for histopathology) was performed by the referring Doctor of Veterinary Medicine (DVM). Ethical approval for the study was granted by the Douglas College Animal Care Committee (accredited by the Canadian Council on Animal Care), and all procedures were performed after obtaining owner consent.

Dental disease was graded (dental score 1‐4) subjectively based on visual examination. Hypertension was defined as SBP > 160 mm Hg measured on at least 2 occasions or on one occasion with hypertensive retinal lesions. Renal azotemia (considered consistent with CKD) was defined as elevated serum creatinine concentration (>2.5 mg/dL [the upper limit of the laboratory (IDEXX Reference Laboratories Ltd, Delta, British Columbia, Canada). RI derived from 173 healthy cats]) in conjunction with poorly concentrated urine specific gravity (USG) (<1.035). Chronic kidney disease (IRIS stage 2) was defined as a serum creatinine concentration of 1.6 to 2.8 mg/dL *plus* USG < 1.035 on at least two occasions plus at least one associated clinical sign: PU/PD, hypertension, UPCR > 0.4, recurrent urinary tract infections, history of acute kidney injury, hypokalemia, hyperphosphatemia, nonregenerative anemia, or weight loss > 5%.

Proteinuria was defined as UPCR > 0.4 without evidence of pyuria or hematuria. Weight loss ≥ 5% in a cat with concurrent disease and >10% in cats without detectable concurrent disease was considered a marker of clinically important concurrent disease in cats fed ad libitum. Feline immunodeficiency virus positive cats were considered to have clinically important illness if they had evidence of inflammatory disease (eg, gingivitis/stomatitis), anemia or leukopenia, or weight loss (or a combination of signs).

Cats were divided into two groups: Group 0 (no clinically important illness detected) and group 1 (noncritically ill) to analyze the effect of illness on the response to exogenous TSH.

### 2.2 Radioiodine I^131^‐treated cats

A total of 949 hyperthyroid cats previously treated with RAI over a 4‐year period (2013‐2016) at a single RAI treatment center were evaluated for inclusion into this prospective study. Cats were excluded if they were suffering from acute/severe illness (hospitalized cats, cats undergoing investigation of acute/severe clinical signs, and cats that required emergency care on the day of their examination for the study), if sedation would likely be required for examination, if they were on a thyroid supplementation or iodine restricted diet or on thyroid‐suppressive medications (see above). All excluded medications had a minimum withdrawal period of 6 weeks. Cats were included in the study at least 12 weeks after RAI treatment.

Owners of eligible cats that met the inclusion criteria were offered a wellness examination and laboratory testing. Data collected for each cat included RAI treatment details, referring DVM medical record, clinical signs questionnaire, physical examination findings (performed by one of two DVMs), SBP measurement, routine biochemistry and hematology, urinalysis and UPCR, thyroid hormone measurements (tT4, fT4, TSH), and TSH stimulation test. Control cats were tested for feline leukemia virus/feline immunodeficiency virus (FIV) status using an ELISA method (IDEXX Laboratories SNAP Combo FeLV Ag/FIV Antibody Immunoassay).

RAI cats were assessed for concurrent illness using the same criteria as used for the control cats (see above) and divided into two groups: no clinically important illness detected and noncritically ill, to analyze the effect of illness on the thyroid hormone results.

RAI cats were also divided into 3 groups (RAI‐hypothyroid, RAI‐euthyroid, and RAI‐equivocal groups) according to their TSH stimulation test results.

### 2.3 Systolic blood pressure measurement

A noninvasive Doppler technique was used to measure SBP after a period of acclimatization, and using previously published methods.^20^ Average SBP was calculated from a minimum of 3 consecutive readings (within 5 mm Hg). If average SBP was ≥160 mm Hg, indirect fundoscopy was performed after applying one drop of tropicamide 1% to both eyes.

### 2.4 Sample collection and handling

All baseline blood samples for thyroid hormone analysis and biochemistry were collected in the morning and placed into serum separator tubes. After the initial blood collection, 0.05 mg of rhTSH was administered IV and a second blood sample was collected 6 hours later for repeat measurement of tT4. Blood was allowed to clot for a minimum of 20 minutes, centrifuged within 1 hour, stored at 4°C to 6°C. Urine was collected by cystocentesis. All samples collected were analyzed within 24 hours at a single local commercial laboratory.

### 2.5 TSH stimulation test

Each 1.1 mg vial of rhTSH (THYROGEN thyrotropin alfa for injection, Genzyme Corp. Thyrotropin alpha lyophilized powder for reconstitution) was reconstituted to a concentration of 1 mg/mL according to the manufacturer instructions, divided into 22 individual doses (0.05 mL) and stored at 4°C to 6°C for a maximum of 4 weeks as previously described.^21^ Dilution and aliquot assignment into individual syringes was done by a compounding pharmacy under sterile conditions.

### 2.6 Biochemical assays

Serum tT4 was measured by a validated^22^ homogenous enzyme immunoassay (DRI Thyroxine (T_4_) assay, Microgenics Corporation, Freemont, California) on an automated biochemistry analyzer (AU2700 Clinical Chemistry System, Beckman Coulter, Brea, California). Serum fT4 was measured using a semiautomated chemiluminescent immunoassay method for measuring fT4 with veterinary‐specific modification (VfT4) (IMMULITE 2000 Veterinary Free T4 (Catalogue no. L2KVF42), Siemens Healthcare Diagnostics Products Ltd. Glyn Rhonwy, Llanberis, Gwynedd LL55 4EL, United Kingdom). TSH in cats was measured using a canine TSH (IMMULITE 2000 Canine TSH (Catalogue no. L2KKT6), Siemens Healthcare Diagnostics Products Ltd. Glyn Rhonwy, Llanberis, Gwynedd LL55 4EL, United Kingdom) assay previously validated with feline samples.^23^ VfT4 and TSH were run on a commercial immunoassay system (Immulite 2000 XPi; Siemens Medical Solutions. Flanders, New Jersey).

Renal function was assessed by measurement of serum creatinine concentrations (by Jaffe reaction), blood urea nitrogen (BUN) concentrations, USG, UPCR, and SBP.

### 2.7 Data presentation and statistical analysis

All statistical analyses were performed using proprietary statistical software (IBM SPSS Statistics 24). All analyses used nonparametric tests due to small group sizes and inconsistent normal distribution of data. All data are presented as median (interquartile range). Statistical significance was defined as *P* < .05. The results of the TSH stimulation test are reported as the serum tT4 concentration 6 hours after IV TSH administration (after‐stimulation tT4) and as the ratio of the tT4 concentration before and after TSH administration (T4‐ratio).

#### 2.7.1 Control cats

Using the control group data, a RI for after‐stimulation tT4 and tT4 ratio was generated using the robust method with Box‐Cox transformation of the data by proprietary software.^24^ The after‐stimulation‐tT4 and tT4 ratio were compared between the two control cat groups using the Mann‐Whitney *U* test with a *P* value <.05 used to indicate significance.

#### 2.7.2 RAI‐treated cats

Continuous variables were compared between groups by the Kruskal‐Wallis test, followed by Dunn's multiple comparisons test. Hormone values below the limit of detection, or above the upper threshold, of the assay were assigned arbitrary values for the analysis as follows: TSH <0.03 = 0.02 ng/mL, TSH >12 = 12 ng/mL, fT4 < 0.29 ng/dL = 0.27 ng/dL. All categorical variables were compared using the Pearson Chi‐squared test, including an assessment of the proportion of cats with and without illness, between the three thyroid function groups.

## 3 RESULTS

### 3.1 Control cats

A total of 36 control cats were recruited, 12 from private homes and 24 group shelter‐housed. Three cats were diagnosed with hyperthyroidism and excluded, therefore 33 cats were included in study. Many shelter cats were of unknown age but all were estimated to be at least 6 years old. Of the 21 cats whose age was known, the median age was 10 years old.^7^, ^8^, ^9^, ^10^, ^11^, ^12^, ^13^, ^14^

The majority of control cats included in the study were Domestic short‐ or long‐haired cats (n = 31) and two purebred cats were included (Persian [n = 1], Peterbald [n = 1]). All cats were neutered with 17 female cats and 16 male cats included in the study. The median weight of control group cats (4.9 [3.7‐6.1] kg) was not significantly different from the RAI‐treated cats (5.0 [4.1‐6.0] kg: *P* = .48; Table 1).

**TABLE 1:** Thyroid hormone concentrations, response to TSH administration and selected biochemical and clinical measurements, in mature adult control cats divided into two groups: apparently healthy (group 0) and noncritically ill (group 1)

Variable	Control group 0	Control group 1	
Weight (kg)	5.25 [3.8‐6.3]	4.55 [3.52‐5.63]	*P* = .15
SBP (mm Hg)	120 [108‐132]	118 [110‐147]	*P* = .88
Heart rate (bpm)	216 [180‐228]	168 [158‐199]	*P* = .003
Creatinine (mg/dL)	1.28 [1.18‐1.81]	1.37 [1.24‐1.67]	*P* = .4
BUN (mg/dL)	28 [21.8‐33.3]	25.2 [20.4‐32.2]	*P* = .71
tT4 (μg/dL)	2.0 [1.72‐2.17]	2.07 [1.61‐2.34]	*P* = .99
fT4 (ng/dL)	1.2 [1.1‐1.6]	1.4 [1.0‐1.9]	*P* = .9
TSH (ng/mL)	0.04 [<0.03‐0.07]	0.03 [<0.03‐0.07]	*P* = .71
After‐stimulation tT4 (μg/dL)	5.2 [4.6‐5.9]	4.7 [3.4‐5.7]	*P* = .38
T4 ratio	2.4 [2.1‐2.9]	2.3 [1.9‐2.8]	*P* = .46

*Note*: Data are presented as median [interquartile range].

Abbreviations: BUN, blood urea nitrogen; SBP, systolic blood pressure; TSH, thyroid stimulating hormone.

Medications administered at the time of the TSH stimulation test included: preventative flea treatments (selamectin (Revolution Zoetis Canada 16740 Trans‐Canada Hwy, Kirkland, Quebec H9H 4J4, Canada) n = 4, imidacloprid/pyriproxyfen (Advantage II Bayer Inc., 2920 Matheson Boulevard East. Mississauga, Ontario Canada L4W 5R6) n = 1), benazepril (Fortekor Elanco 150 Research Lane, Suite 120 Guelph, ON N1G 4T2) and ranitidine (Zantac® Sanofi 2905 Place Louis‐R.‐Renaud, Laval, QC H7V 0A3, Canada) (n = 1), potentiated amoxicillin (Clavaseptin Vetoquinol N.‐A inc 2000, chemin Georges, Lavaltrie, Québec J5T 3S5, Canada) (n = 1), insulin (Lantus® Sanofi 2905 Place Louis‐R.‐Renaud, Laval, QC H7V 0A3) (n = 1).

Control cats in group 0 (no clinically important illness: n = 15) included cats with mild to moderate dental calculus or isolated suspect feline odontoclastic resorptive lesions (n = 10), cats with a grade 2 to 3 heart murmur but no other evidence of heart disease (n = 2), and an apparently healthy FIV positive cat (n = 1).

Control cats in group 1 (noncritically ill: n = 18) included a mildly jaundiced cat with early evidence of hepatic lipidosis (hepatic lipidosis later confirmed by biopsy), FIV positive cats with moderate‐severe gingivitis/stomatitis, weight loss, or both (n = 3), a cat with 3rd degree heart block, severe hypertension (SBP > 200 mm Hg) and CKD (IRIS stage 2), a treated but poorly controlled diabetic cat with a heart murmur, cats with IRIS stage 2 CKD (n = 2), a nonpyrexic cat with evidence of marked systemic inflammation/infection including bands and toxic neutrophils, cats with grade 4 dental disease and evidence of oral pain (n = 2) and cats with multiple clinically important markers of illness such as moderate hypokalemia (K^+^ 3.0‐3.5 mmoL/L; n = 2), moderate nonregenerative anemia (PCV = 20%: n = 1), hypoalbuminemia (albumin = 22 g/L: n = 1), systolic hypertension (n = 3), and proteinuria (n = 1). Weight loss was recorded in 12/18 of the cats in this group, 3 cats had stable or increased weight, and 3 cats had no historical weight data available.

### 3.2 TSH stimulation test results in control group cats

The T4 ratio (*P* = .46) and after‐stimulation‐tT4 (*P* = .38) were not significantly different between the two control cat groups (Table 1).

The control cat data were used to derive a RI minimum after‐stimulation tT4 concentration (2.3 μg/dL) and a reference minimum tT4 ratio (1.5) after rhTSH administration. These values were used to determine the euthyroid cut‐off points for the RAI‐treated cats.

### 3.3 TSH stimulation test criteria for RAI‐treated cats

**FIGURE 1:**
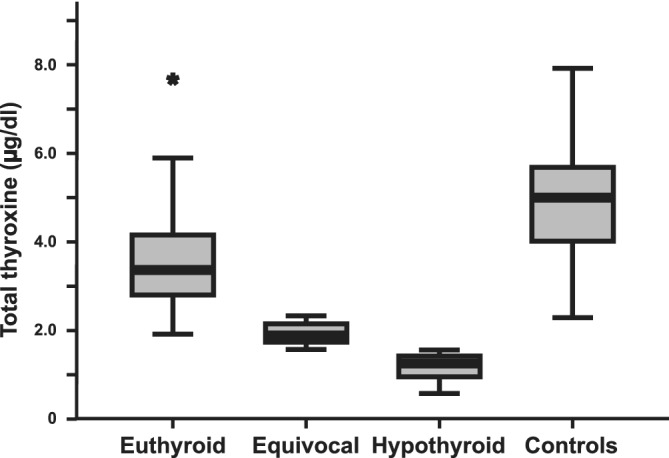
Boxplot demonstrating the concentration of total thyroxine (tT4), 6 hours after intravenous rhTSH administration, in 4 groups of cats (after‐stimulation tT4). The 4 groups are RAI‐*Euthyroid* (RAI‐treated cats with good response to rhTSH administration n = 71), RAI‐*Equivocal* (RAI‐treated cats with poor response to rhTSH administration n = 21), RAI‐*Hypothyroid* (RAI‐treated cats with low or low normal tT4 concentration and no, or inadequate, response to rhTSH administration n = 26) and Controls (euthyroid mature adult cats n = 33). The box in the boxplot represents the interquartile (IQ) range, the horizontal bar represents the median value. The whiskers represent the data outside the IQ range but excluding individual values >1.5 box‐lengths from the upper and lower edges of the box. Outliers >1.5 box‐lengths from the edges of the box are represented by stars. RAI, radioiodine‐I^131^; rhTSH, recombinant human thyroid stimulating hormone

**FIGURE 2:**
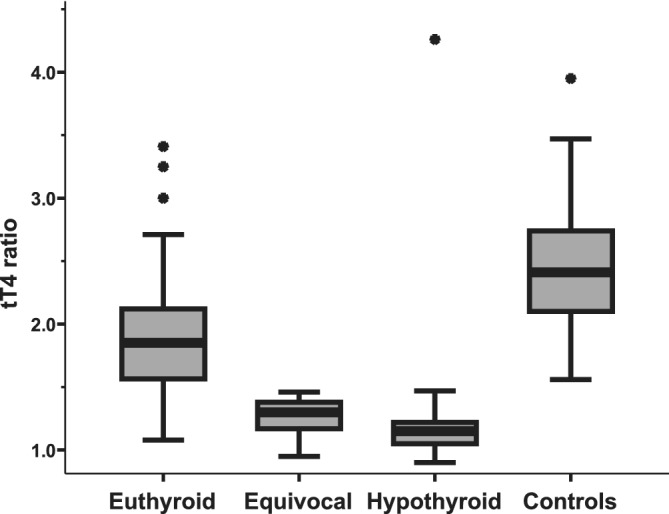
Boxplot demonstrating the ratio of total thyroxine concentration (tT4 ratio), before and 6 hours after intravenous rhTSH administration, in 4 groups of cats. Control cats had a significantly higher (*P* < .001) T4 ratio compared to the radioiodine treated cats. The groups are as described in Figure 1 legend. See Figure 1 for boxplot key. rhTSH, recombinant human thyroid stimulating hormone

After administration of rhTSH, the control group cats had a higher median after‐stimulation tT4 (5.0 [3.9‐5.8] μg/dL) and T4ratio (2.41 [2.09‐2.82] μg/dL) than the RAI‐treated cats (Figures 1 and 2: *P* < .001).

The RAI‐treated cats were divided into three groups based on the following criteria: RAI‐euthyroid group (n = 71); after‐stimulation tT4 ≥ 2.3 μg/dL *or* after‐stimulation tT4 1.5 to 2.3 μg/dL *and* before : after tT4 ratio ≥ 1.5, RAI‐equivocal group (n−21); after‐stimulation tT4 1.5 to 2.3 μg/dL *and* tT4 ratio < 1.5, RAI‐hypothyroid group (n = 26); after‐stimulation tT4 ≤ 1.5 μg/dL.

### 3.4 RAI‐treated cats

A total of 118 RAI‐treated cats were recruited including 106 domestic short‐ or long‐haired cats and 12 purebred cats: all cats were neutered (female n = 64, male n = 54). Twenty cats were receiving medication at the time of the TSH stimulation test including potassium supplements (n = 6), insulin (n = 2), cyclosporine (Lantus Sanofi 2905 Place Louis‐R.‐Renaud, Laval, QC H7V 0A3, Canada) (n = 1), benazepril (n = 2), maropitant citrate (Atopica Elanco 150 Research Lane, Suite 120 Guelph, ON N1G 4T2, Canada) (n = 1) famotidine (Cerenia Zoetis Canada 16740 Trans‐Canada Hwy, Kirkland, Quebec H9H 4J4) (n = 2) and the following generic or compounded drugs: amlodipine (n = 3), buprenorphine (n = 1), gabapentin (n = 2), atenolol (n = 1), ursodiol (n = 3), and amoxicillin (n = 1). Radioiodine I^131^‐treated cats were older (14 [12–15] years) than the control cats (9 [7.0‐10.5] years: *P* < .001) (Table 2).

**TABLE 2:** RAI treatment variables and select clinical and biochemical variables in mature adult euthyroid cats, and in RAI‐treated cats divided into three groups according to response to TSH administration (see Section 2)

Variable	Control group	RAI‐euthyroid	RAI‐equivocal	RAI‐hypothyroid	
Number (n)	33	71	21	26	
Age (y)	9 [7.0‐10.5]^1,2,3^	13 [11‐15]^1,4^	14 [11.5‐15.5]^2^	15 [14‐16]^3,4^	^1,2,3^ *P* < .001 ^4^ *P* = .01
Weight (kg)	4.9 [3.7‐6.0]	5.3 [4.4‐6.5]^1^	4.3 [3.9‐5.4]	4.2 [3.8‐5.7]^1^	^1^ *P* = .009
SBP (mm Hg)	119 [108‐134] ^1,2^	133 [121‐149]^1^	140 [115‐150]	150 [123‐166]^2^	^1^ *P* = .005 ^2^ *P* = .02
Heart rate (bpm)	192 [161‐216]	180 [168‐192]	180 [174‐192]	171 [154‐192]	
Creatinine (mg/dL)	1.36 [1.20‐1.76]^1,2,3^	1.85 [1.49‐2.33]^1,4^	1.76 [1.39‐1.96]^2,5^	2.3 [1.83‐2.92]^3,4,5^	^1,3^ *P* < .001 ^2^ *P* = .02 ^4,5^ *P* = .003
BUN (mg/dL)	26.6 [21.6‐32.8]^1^	29.1 [26.6‐35.3]^2^	31.1 [20.7‐44.2]	33.3 [28.6‐45.4]^1, 2^	^1,^ *P* = .001 ^2^ *P* = .02
RAI dose (miCu)	NA	3.5 [2.5‐4.1]	3.2 [2.35‐4.2]	3.65 [2.65‐4.38]	
Before RAI tT4 (μg/dL)	NA	8.9 [6.1‐12.4]	9.5 [5.6‐13.3]	11.1 [5.9‐13.2]	
Time to T0 (d)	NA	427 [298‐784]	274 [212‐546]	435 [219‐563]	

*Note*: Data are presented as median [interquartile range]. Values with the same superscript numbers are significantly different to one another.

Abbreviations: BUN, blood urea nitrogen; RAI, radioiodine I^131^; SBP, systolic blood pressure; TSH, thyroid stimulating hormone.

Fifty‐one of the 118 RAI cats were identified as having noncritical illness. This included azotemia (n = 25), nonazotemic IRIS grade 2 CKD (n = 15), grade 4 dental disease (n = 6, of which 4 also had CKD), untreated hypertension (n = 9, of which 4 also had CKD) or other concurrent disease/weight loss (diabetes mellitus n = 2, gastrointestinal disease n = 2, hepatitis n = 1, heart disease n = 2).

There was no significant difference in the proportion of cats with and without illness between the hypothyroid and nonhypothyroid groups (*P* = .34) and no significant difference when the effect of illness on after‐stimulation tT4 was compared within the 3 (RAI‐euthyroid, RAI‐intermediate, and RAI‐hypothyroid) groups (*P* = .54).

### 3.5 Thyroid hormone concentrations

The median TSH concentration in control cats was significantly lower (0.04 [<0.03‐0.07] ng/mL) than the median TSH in the RAI‐euthyroid group (0.13 [0.07‐0.24] ng/mL: *P* < .001). Control cats also had a significantly higher fT4 (1.3 [1.1‐1.6] ng/dL) compared to the RAI‐euthyroid group (1.1 [0.9‐1.4] ng/dL: *P* = .04), however, no difference in tT4 concentrations was observed (Table 3).

**TABLE 3:** Thyroid hormones at baseline and after administration of rhTSH in control cats and in RAI‐treated cats divided into three groups according to response to TSH administration (see Section 2)

Variable	Control group	RAI‐euthyroid	RAI‐equivocal	RAI‐hypothyroid	
tT4 (μg/dL)	2.0 [1.7‐2.3] ^1,2^	1.8 [1.6‐2.1] ^3,4^	1.6 [1.4‐1.7]^1,5^	1.0 [0.8‐1.3]^2^	^1,2,3,4,5^ *P* < .001
fT4 (ng/dL)	1.3 [1.1‐1.6] ^1,2,3^	1.1 [0.9‐1.4] ^1,4,5^	0.9 [0.6‐1.1] ^2,4,6^	0.35 [0.27‐0.56] ^3,5,6^	^1^ *P* = .04 ^2,3,4,5,6^ *P* < .001
TSH (ng/mL)	0.04 [<0.03‐0.07] ^1,2,3^	0.13 [0.07‐0.24] ^1,4,5^	0.31 [0.17‐0.58] ^2,4,6^	1.4 [0.39‐5.4] ^3,5,6^	^1,2,3,5^ *P* < .001 ^4^ *P* = .002 ^6^ *P* = .003
After‐stimulation tT4 (μg/dL)	5.0 [3.9‐5.8] ^1,2,3^	3.4 [2.8‐4.2] ^1,4,5^	1.9 [1.7‐2.1] ^2,4,6^	1.2 [0.9‐1.5]^3,5,6^	^1,2,3,4,5,6^ *P* < .001
T4 ratio	2.41 [2.09‐2.82]^1,2,3^	1.85 [1.56‐2.13] ^1,4,5^	1.30 [1.16‐1.38]^2,4,6^	1.15 [1.04‐1.23] ^3,5,6^	^1,2,3,4,5^ *P* < .001 ^6^ *P* = .003

*Note*: Data are presented as median [interquartile range]. Values with the same superscript numbers are significantly different to one another.

Abbreviations: RAI, radioiodine‐I^131^; rhTSH, recombinant human thyroid stimulating hormone; TSH, thyroid stimulating hormone.

RAI‐hypothyroid cats had significantly higher TSH (*P* < .001), lower tT4 (*P* < .001), and lower fT4 (*P* < .001) than the RAI‐euthyroid or RAI‐equivocal groups (Table 3).

**FIGURE 3:**
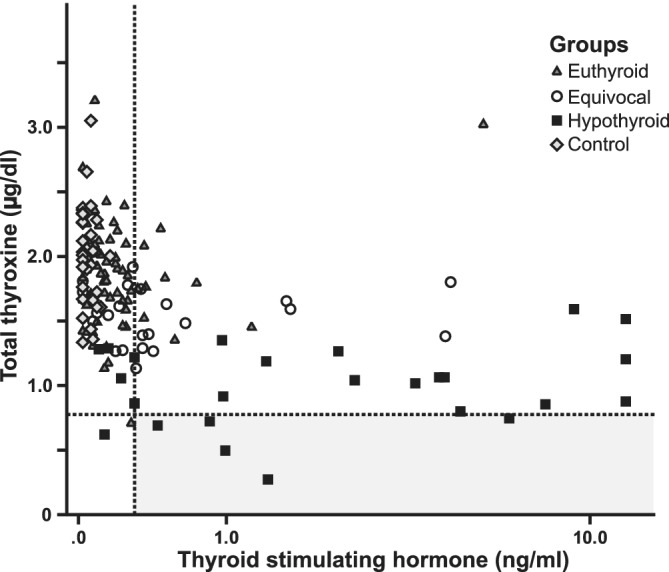
Scatterplot of baseline total thyroxine and endogenous TSH concentrations in RAI‐treated cats divided into three groups based on response to exogenous rhTSH (groups described in Figure 1.). The horizontal dotted line represents the lower end of the laboratory reference range for tT4 and the vertical dotted line represents a previously published^6^ reference cut‐off for TSH in cats (0.3 ng/mL). The gray box depicts the diagnostic area for hypothyroidism if both tT4 and TSH concentrations were to be used for diagnosis, and using the published reference cut‐offs. RAI, radioiodine‐I^131^; rhTSH, recombinant human thyroid stimulating hormone; TSH, thyroid stimulating hormone

**FIGURE 4:**
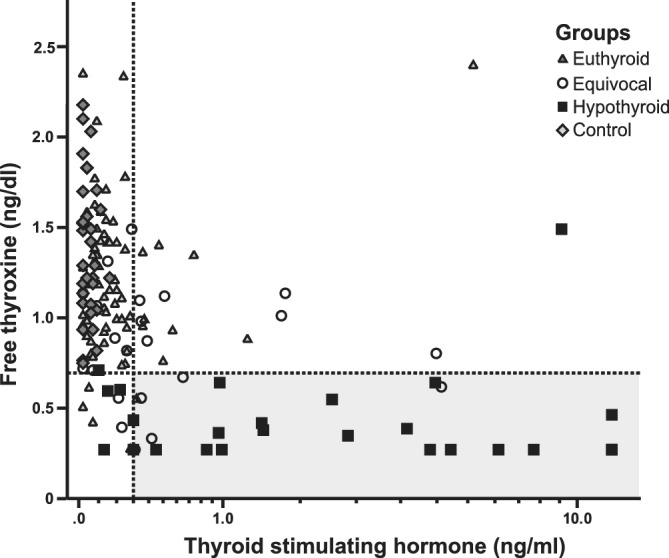
Scatterplot of baseline free thyroxine and endogenous TSH concentrations in RAI‐treated cats divided into three groups based on response to exogenous rhTSH (groups described in Figure 1). Control cats are included for comparison. The horizontal dotted line represents the lower end of the laboratory reference range for fT4 and the vertical dotted line represents a previously published^6^ reference cut‐off for TSH in cats. The gray box depicts the diagnostic area for hypothyroidism if both fT4 and TSH concentrations were to be used for diagnosis, and using published reference cut‐offs. RAI, radioiodine‐I^131^; rhTSH, recombinant human thyroid stimulating hormone; TSH, thyroid stimulating hormone

Only 5/26 (19%) RAI‐hypothyroid cats in our study had a tT4 concentration below the laboratory RI (<0.78 μg/dL; Figure 3); however, low fT4 (<0.70 ng/dL) was detected in 88% of the RAI‐hypothyroid cats (Table 4). TSH was high in (85%) of RAI‐hypothyroid cats but 14% of the RAI‐euthyroid and 57% of the RAI‐equivocal groups also had high TSH. Of the 5 RAI‐euthyroid cats with low fT4, only 1/5 had a high TSH (0.32 ng/mL, Figure 4).

**TABLE 4:** Proportion (number) of RAI‐treated cats with fT4, and tT4 below the laboratory reference range, and TSH > 0.3 ng/mL when divided into RAI‐hypothyroid, RAI‐equivocal, and RAI‐euthyroid groups using the TSH stimulation test results

Thyroid hormone	RAI‐hypothyroid % (n)	RAI‐equivocal % (n)	RAI‐euthyroid % (n)
Number of cats	26	21	71
fT4 < 0.70 ng/dL	88% (23)	33% (7)	7% (5)
tT4 < 0.78 μg/dL	19% (5)	0% (0)	1.4% (1)
TSH ≥ 0.3 ng/mL	85% (22)	57% (12)	14% (10)

Abbreviations: RAI, radioiodine‐I^131^; TSH, thyroid stimulating hormone.

### 3.6 Relationship between thyroid status and radioiodine treatment

The median [IQ range] dose of RAI received by the RAI‐treated cats was 3.5 [2.5‐4.1] mCi (Table 2) and the majority of cats were included in the study at least 12 months (422.5 [269‐697] days) after RAI treatment. In cases where two RAI treatments were given, the second dose is included in the data set and the date of the second treatment used to calculate time after treatment. The median tT4 concentration at diagnosis (before RAI treatment) in the RAI‐treated cats was 9.1 (6.1‐12.7) μg/dL. There were no significant differences in the radioiodine dose received (*P* = .41), highest before‐treatment tT4 (*P* = .83), or time interval between RAI treatment and TSH stimulation test (*P* = .22), between the three groups (Table 2).

### 3.7 Relationship between thyroid status and renal function

RAI‐hypothyroid cats were significantly more likely to be azotemic (39%) than the RAI‐euthyroid (14%) and RAI‐equivocal (15%) groups (*P* = .03) and had a higher serum creatinine concentration (2.30 [1.83‐2.93] mg/dL) than the RAI‐equivocal (1.76 [1.39‐1.96] mg/dL]; *P* = .003) or RAI‐euthyroid (1.85 [1.49‐2.33] mg/dL; Table 2; *P* = .003) cats. RAI‐hypothyroid cats also had a higher BUN (33.3 [28.6‐45.4] mg/dL) than the RAI‐euthyroid (29.1 [26.6‐35.3] mg/dL) cats (*P* = .02). There was no significant difference in USG or UPCR between the three groups.

### 3.8 Relationship between thyroid status and SBP

Cats receiving amlodipine treatment at the time of examination were not included in the analysis of blood pressure (n = 4; all in the RAI‐euthyroid group). There was no significant difference in SBP between the three RAI‐treated groups; however, SBP was higher in the RAI‐hypothyroid group compared to the control group (*P* = .02).

## 4 DISCUSSION

The principal findings of the study are that noncritical illness does not appear to affect the results of the TSH stimulation test in mature adult cats, and that the response to TSH stimulation is typically suppressed in RAI‐treated cats compared to controls. The proportion of RAI‐hypothyroid cats with a low fT4 concentration (88%) and a high TSH concentration (85%) was high, but a high proportion of RAI‐equivocal (57%) and RAI‐euthyroid (14%) also had high TSH. Therefore, use of TSH alone as a diagnostic test for hypothyroidism could lead to a high risk of false positive diagnoses. Conversely, if a clinician were to rely solely on a tT4 concentration below the RI to identify possible hypothyroidism in RAI‐treated cats, there is a high risk of a false negative diagnosis, that is, a high proportion (81%) of hypothyroid cats would be missed.

Two recent studies of RAI‐treated cats have used scintigraphy as the reference test to determine thyroid function. In the first study of iatrogenic hypothyroidism in RAI‐treated cats with CKD,^6^ TSH had a high sensitivity and specificity, and fT4 had a low sensitivity, for the diagnosis of iatrogenic hypothyroidism. In the second study,^13^ which did not identify any hypothyroid cats on scintigraphy, 27% of euthyroid cats had a high TSH, and 41% euthyroid cats had a low fT4 (using the same non‐ED assay). In contrast, 7% of RAI‐euthyroid cats in our study had low fT4, and 14% had high TSH (furthermore RAI‐equivocal cats had higher proportions with low fT4/high TSH).

There could be a number of reasons for the difference between the findings of our study and previous studies. The TSH stimulation test, scintigraphy, and histopathology have all been used as reference tests for the diagnosis of primary hypothyroidism in dogs. One dog study concluded that scintigraphy is more reliable than the TSH stimulation test to distinguish nonthyroidal illness from hypothyroidism, in dogs with a low tT4.^14^ In addition, iodine trapping can be decreased or asymmetrical in dogs with various nonthyroidal illnesses, and technetium uptake by the thyroid can be affected by some drugs, amyloidosis, high iodine content in the diet and active thyroiditis.^25^, ^26^ There are no similar studies comparing the reliability of these reference tests in cats. Hypothyroidism in dogs has a different etiopathogenesis and pathodynamics compared with iatrogenic hypothyroidism in cats, therefore extrapolation of the findings of dog studies to cats might not be valid. One of the previous studies of iatrogenic hypothyroidism in RAI‐treated cats only included cats with CKD, whereas our study recruited any RAI‐treated cat that met our inclusion criteria, regardless of renal function. Since CKD can change the dynamics of iodine metabolism and excretion,^27^ it is possible that technetium uptake could be altered in cats with CKD compared to cats with normal renal function, therefore affecting the reliability of thyroid scintigraphy as a diagnostic test for iatrogenic hypothyroidism in cats with CKD.

Our study used the TSH stimulation test as a reference test of thyroid function in RAI‐treated cats. The cut‐points used to interpret the TSH stimulation test results in our study were based on RIs derived from a group of mature euthyroid adult control cats. The TSH stimulation test is a test of thyroid reserve and it is apparent from our data set that the majority of RAI‐treated cats had a decreased thyroid reserve compared to untreated euthyroid cats. As with all endocrine diseases, it is hard to place defined cut‐points into a system where many individuals fall into a gray zone between clearly normal and clearly abnormal, and we therefore included a 3rd group of cats with “equivocal” function. This approach has been used previously, for example, in studies of thyroid function in dogs^15^ and in a recent study of diagnostic tests for pancreatitis in cats.^28^ The inclusion of this third group of cats meant that a calculation of sensitivity and specificity for thyroid hormones as diagnostic tests for hypothyroidism could not be performed in our study.

Our study did not find a difference in response to TSH between control cats with noncritical illness and relatively healthy controls, or between the different groups of RAI‐treated cats, which concurs with the findings of a previous study.^18^ As noted above, sick euthyroidism can lead to a decreased response to exogenous TSH administration, with the potential for a false positive diagnosis of hypothyroidism.^14^, ^29^ It was therefore important for us to determine if the suppressed response to TSH in our study was due to concurrent illness, before we attributed the results to a depressed thyroid reserve. There were a wide variety of different types of concurrent illnesses in our study cats, which is common in senior cat populations. Although we were able to apply objective measures of illness in some cases (eg, blood pressure and weight loss), in some cases the categorization of cats as “ill” and “not ill” somewhat subjective (eg, the assessment of dental disease). It is important to note that our recruitment strategy resulted in the inclusion of a relatively healthy population of cats and none of our control cats had subnormal tT4 despite their illnesses. Therefore, our conclusion that noncritical illness had no effect on the results of the TSH stimulation test cannot be extrapolated to sick euthyroid cats with subnormal tT4 concentrations.

High TSH (>0.3 ng/mL) has a 98.5% specificity and a 100% sensitivity for the diagnosis of hypothyroidism^6^ whereas in our study, 85% of RAI‐hypothyroid cats had high TSH (suggesting a lower sensitivity) and 57% of the RAI‐equivocal cats had high TSH (suggesting a lower specificity). Our results also concur with another study in which the specificity of high TSH for hypothyroidism was 73%.^13^ This difference in the sensitivity and specificity of TSH is of particular note because the same canine TSH assay was used in all three studies. It could be speculated that these differences could be due to the criteria we used to define hypothyroidism and euthyroidism. For example, it is possible that some RAI‐equivocal and even RAI‐euthyroid cats in our study might have been categorized as hypothyroid using scintigraphy. In order to be categorized as euthyroid for our study, RAI‐treated cats did not have to meet a minimum 50% increase in tT4 after‐TSH administration, if their after‐stimulation tT4 was >2.3 μg/dL. I^131^RAI‐euthyroid cats in our study had a higher median TSH than the control group and 10/71 cats in the RAI‐euthyroid group had a high TSH (≥0.3 ng/mL); however, only 3 of the 10 euthyroid cats with high TSH and after‐stimulation tT4 > 2.3 μg/dL had a tT4 ratio < 1.5.

Previous studies have shown that pituitary secretion of TSH takes 1 to 3 months to recover after resolution of hyperthyroidism^5^; however all cats in our study were tested >3 months after RAI with a median time of 422 days between RAI and the TSH stimulation test. In addition, there was no difference in time interval between RAI and TSH stimulation test in the RAI‐euthyroid, RAI‐equivocal and RAI‐hypothyroid cats. Therefore, the timing of the TSH stimulation test in relation to RAI treatment is unlikely to have influenced the categorization of the RAI cats into different thyroid function groups.

The non‐ED veterinary free T4 assay used in this study correlated better with a diagnosis of hypothyroidism compared to the ED fT4 assay used in the Peterson study^6^. In the Peterson study, low fT4 (below the laboratory RI) had a sensitivity and specificity of 25% and 98% respectively, whereas in our study, 88% of RAI‐hypothyroid cats had a fT4 below the laboratory RI. In a separate study, which used the same method and fT4 cut‐point (0.7 ng/dL) for diagnosis of hypothyroidism as our study, 41% of euthyroid cats had a fT4 below the laboratory RI, whereas in our study 7% of RAI‐euthyroid and 33% of RAI‐equivocal had a low fT4.^13^ These data suggest that the non‐ED veterinary fT4 assay used in our study (and the study by Stameleer et al) gives fewer false negative results (is more sensitive) but more false positive results (is less specific) for iatrogenic hypothyroidism in cats than the assay used in the Peterson study. This could reflect differences in assay calibration or assay sensitivity that leads to lower measured fT4 concentrations with our assay. This supposition is supported by our data set in which >25% of RAI‐hypothyroid cats had a fT4 below the limit of detection, whereas most of these cats had detectable tT4 concentrations. Although a high correlation between the non‐ED fT4 assay used in our study and tT4 has been reported,^13^ the aforementioned study did not perform a Bland Altman analysis to test for systematic differences between these variables. Therefore, it is possible that the non‐ED fT4 assay used in both the aforementioned study and our study consistently underestimates the fT4, which could account for the lower false negative and higher false positive rate observed in our study compared to the Peterson study. In addition, only one of the 71 RAI‐euthyroid cats had both a low fT4 (0.55 ng/dL) and a high TSH (0.32 ng/mL), which suggests that combining fT4 and TSH measurements might decrease the risk of a false positive hypothyroidism diagnosis compared to using fT4 or TSH alone.

The non‐ED fT4 assay is a veterinary specific chemiluminescent assay but does not use an ED step to isolate the free (unbound) thyroxine from the protein‐bound fraction. A number of studies have looked at the reliability of fT4 assays^11^, ^12^ for the diagnosis of hypothyroidism and hyperthyroidism, using both radio‐assay and chemiluminescent methods, with and without ED, and most studies have concluded that ED methods are superior to non‐ED methods. Free T4 could not be determined by ED in our study due to financial constraints, therefore we could only evaluate the performance of the commercial veterinary chemiluminescent free T4 assay available at our local reference laboratory. Our results suggest that this fT4 assay (particularly in combination with TSH) might be a reliable test for iatrogenic hypothyroidism in cats, when TSH stimulation testing is used as the reference test; however, additional validation studies are required to confirm analytical precision and reproducibility.

As with previous studies in cats,^6^, ^18^ baseline tT4 concentrations were within reference range in the majority of cats diagnosed with iatrogenic hypothyroidism. This reinforces the need to measure TSH and fT4 when assessing RAI‐treated cats for biochemical evidence of hypothyroidism, and veterinarians need to be especially vigilant in RAI‐treated cats with low‐normal tT4 concentrations (0.78‐1.5 μg/dL). In this study, none of the RAI‐equivocal, and only one RAI‐euthyroid cat had a tT4 below the RI (<0.78 μg/dL), likely due to the fact that our study design and inclusion/exclusion criteria excluded cats likely to have severe nonthyroidal illness. We expect that the specificity of low tT4 for iatrogenic hypothyroidism would be lower in cats with severe nonthyroidal illness, and further studies would be required to confirm this.

In our study, baseline tT4 was lower and response to rhTSH administration was decreased in the euthyroid control cats compared to previous studies.^17^, ^18^, ^19^ It is possible that these differences in baseline T4 and response to exogenous TSH could be due to the much younger age of the cats in the previous studies (7‐8 months and 2 years, respectively), compared with the median age of 9 [7‐10.5] years old of the control cats in our study. There is conflicting evidence that tT4 concentrations change with age in cats, with an effect of age being reported in some studies^30^ but not in others.^9^

In our prospective study, iatrogenic hypothyroidism was common in RAI‐treated cats: 22% of recruited cats were diagnosed with hypothyroidism, with a further 18% of cats in the RAI‐equivocal group demonstrating a subnormal response to rhTSH administration. The high prevalence of elevated endogenous TSH in our study (37%), alongside within‐RI tT4 concentrations, supports the conclusion of a previous study that “subclinical” hypothyroidism appears to be common in RAI‐treated cats.^6^ Despite the fact that tT4 concentrations were within reference range in the majority of our RAI‐treated cats, clinically important effects on kidney function can be inferred due to the significantly higher serum creatinine and BUN concentrations and higher SBP in hypothyroid cats, compared to the control group or euthyroid RAI‐treated cats. Previous studies have demonstrated the link between increased risk of azotemia and hypothyroidism in cats following RAI treatment^5^, ^31^ and a significant effect of untreated hypothyroidism on the survival of RAI and medically treated cats with CKD.^5^, ^6^ Therefore, the identification and treatment of hypothyroidism has important implications on the longevity of RAI‐treated cats.

In our study, SBP was significantly higher in RAI‐hypothyroid cats compared to control cats. In humans, naturally occurring hypothyroidism is associated with an increased prevalence of hypertension, with treatment of hypothyroidism leading to a reduction in systolic and diastolic blood pressure.^32^ The pathophysiological mechanism for hypertension in hypothyroidism is unclear; however, it is postulated that hypertension could be secondary to increased total peripheral resistance, increased β‐adrenergic responsiveness, or extracellular fluid volume expansion.^32^ Therefore, the increased SBP observed in hypothyroid cats in our study could reflect these pathophysiological changes in cats with iatrogenic hypothyroidism. Treatment of hyperthyroidism is associated with the development of systolic hypertension in approximately 23% of initially normotensive hyperthyroid cats (Morrow L, Adams VJ, Elliott J, Syme H. Hypertension in hyperthyroid cats: prevalence, incidence and predictors of its development. J Vet Int Med. 2009;23:700 (abstract)) however to date, no studies have investigated if this correlates with the development of iatrogenic hypothyroidism after treatment. In one previous study, total T4 was not significantly different between treated hyperthyroid cats which were hypertensive or normotensive after treatment; however, serum TSH concentrations were not measured contemporaneously to exclude low TT4 secondary to nonthyroidal illness.^33^

## CONFLICT OF INTEREST DECLARATION

In‐kind support of this study was provided by IDEXX Laboratories in the form of no‐charge laboratory testing: all blood and urine samples collected during the course of the study were analyzed at IDEXX laboratories at no charge. IDEXX Laboratories staff were not involved in the study design, data collection, interpretation of data, or the writing of the report (other than laboratory methods section).

## 5 OFF‐LABEL ANTIMICROBIAL DECLARATION

Authors declare no off‐label use of antimicrobials.

## Institutional Animal Care and Use Committee (IACUC) or Other Approval Declaration

This work involved the use of non‐experimental (owned) animals only. Ethical approval for the study was granted by the Douglas College Institutional Animal Care Committee (accredited by the Canadian Council on Animal Care). Informed Consent was obtained in writing from the owner or legal custodian of all animals described in this work for the procedures undertaken. No animals or humans are identifiable within this publication, and therefore additional Informed Consent for publication was not required.

## 6 HUMAN ETHICS APPROVAL DECLARATION

Authors declare human ethics approval was not needed for this study.
